# Brain Organoids: Studying Human Brain Development and Diseases in a Dish

**DOI:** 10.1155/2021/5902824

**Published:** 2021-09-09

**Authors:** Jie Xu, Zhexing Wen

**Affiliations:** ^1^The Graduate Program in Genetics and Molecular Biology, Laney Graduate School, Emory University, GA 30322, USA; ^2^Department of Psychiatry and Behavioral Sciences, Emory University School of Medicine, Atlanta, GA 30322, USA; ^3^Department of Cell Biology, Emory University School of Medicine, Atlanta, GA 30322, USA; ^4^Department of Neurology, Emory University School of Medicine, Atlanta, GA 30322, USA

## Abstract

With the rapid development of stem cell technology, the advent of three-dimensional (3D) cultured brain organoids has opened a new avenue for studying human neurodevelopment and neurological disorders. Brain organoids are stem-cell-derived 3D suspension cultures that self-assemble into an organized structure with cell types and cytoarchitectures recapitulating the developing brain. In recent years, brain organoids have been utilized in various aspects, ranging from basic biology studies, to disease modeling, and high-throughput screening of pharmaceutical compounds. In this review, we overview the establishment and development of brain organoid technology, its recent progress, and translational applications, as well as existing limitations and future directions.

## 1. Introduction

Being the control center of the nervous system in humans, the brain is one of the most complex and advanced organs in the body, and thus, it has never been easy to study the biological basis of brain development and brain disorders. The current knowledge of the human brain is mostly based on postmortem brain specimens, mainly due to the difficulties in accessing human brain tissues. As a result, animal models, including nonhuman primates, have been widely used to study the development and function of the brain for many decades. However, the human brain differs from those of other species not only in size, shape, and structure but also in cellular and molecular composition and developmental trajectory [1–5]. Hence, a model system that can better recapitulate human brain development is urgently needed to deepen our understanding in human-specific developmental processes and molecular mechanisms.

The advent of stem cell technology has opened a new avenue to study human brain development *in vitro*, providing new platforms for modeling neurological disorders, especially those involves developmental processes that are unique to human [6–8]. For the last decade, human stem cells, including embryonic stem cells (ESCs) and induced pluripotent stem cells (iPSCs), have been widely used in the differentiation of monolayer neural cells to investigate the cellular and molecular mechanisms of neurodevelopment and neurological disorders. While monolayer (two-dimensional) cell culture has provided a system that can efficiently produce relatively homogeneous population of a cell type, they still cannot recapitulate many characteristic features of the human brain, such as self-organizing properties and interactive dynamics [9, 10].

These limitations inspired the innovation of a more sophisticated model system and thus led to the invention of brain organoids. Brain organoids are stem-cell-derived 3D suspension cultures that are capable of self-assembling into an organized structure with features resembling the developing brain, such as ventricle formation, cortical layer organization, and neuronal migration [11–18]. Transcriptomic and epigenomic analysis also revealed that brain organoids recapitulate many features of early-to-mid and mid human fetal brain [19]. Additionally, whereas monolayer cultures can only be maintained for a short period of time, long-term culturing of brain organoids promotes further maturation and thus provides opportunities for investigating late-stage developmental events such as gliogenesis, neuronal maturation, and neuronal network formation. For example, high-depth bulk and single-cell RNA-sequencing confirmed the presence of astrocyte-lineage cells in human cortical spheroids and that these astrocytes resemble primary human fetal astrocytes [18]. Moreover, cerebral organoids cultured for eight months exhibited spontaneously active neurons and neuronal networks and generated photosensitive cells that can respond to light stimulation [20]. Most recently, a comprehensive assessment on the maturation of human cortical organoids reported attainment of early postnatal features when cultured for 250~300 days *in vitro*, which was in a timeline paralleling *in vivo* development. These features included switches in the histone deacetylase complex and NMDA receptor isoform, as well as the emergence of superficial layer neurons and astrocytes at later stages [21].

With the application of diverse advanced technologies such as genome editing, single-cell sequencing, biomaterials, and bioengineering, progress has been made in brain organoids to better recapitulate features of the human brain, including supplementation of brain-blood barrier, vasculature, and microglia. Here, we summarize some of the recent innovations on brain organoid techniques and review the use of human brain organoids on the investigation of neurological and neurodevelopmental disorders as well as potential treatments (Table 1). At the end, we also discuss the limitations of organoid models and highlight potential improvements that would allow brain organoids to progress further in the future.

## 2. Main Text

### 2.1. Current Methodologies of Generating Three-Dimensional Brain Organoids

In general, protocols for induction of brain organoids from stem cells can be classified into two main categories: unguided methods that make use of the spontaneous morphogenesis and intrinsic signaling potential of human pluripotent stem cell (hPSC) aggregates to generate brain organoids that contain a variety of cell lineage identities [14, 22, 23], as well as guided methods that induce regional cell fate specification by applying patterning factors to the culture and lead to the production of brain region-specific organoids [11, 13, 24, 25]. Unguided brain organoids are advantageous in that they have the capacity to develop into various kinds of cell lineages, including dorsal forebrain, ventral forebrain, midbrain, hindbrain, hippocampus, retina, choroid plexus, and even nonneural lineages [14, 20, 22]. Single-cell transcriptomic analyses revealed the presence of neural progenitors, excitatory neurons, inhibitory neurons, astrocytes, oligodendrocyte precursor cells, and photosensitive cells in unguided cerebral organoids, confirming the heterogeneous cellular population of these organoids [20, 26–29]. However, as every coin has two sides, the stochastic nature of hPSC spontaneous differentiation also leads to problems such as unpredictable proportion and arrangement of each cell lineage in the unguided cerebral organoids. Although the variety of cell lineages present in the unguided brain organoids has provided a unique opportunity for examining interactions between different brain regions, the high variability across batches and cell lines has made systematic and quantitative studies difficult and challenging and thus prompts interest in generating brain region-specific organoids through guided differentiation.

The principle of guided differentiation is to utilize small molecules and growth factors to promote a certain cell lineage, forming cells and structures representative of a specific brain region. Typically, neural lineages are promoted by the inhibition of the BMP/TGF-*β* signaling pathway; with subsequent application of relevant patterning factors (i.e., WNT3A, SHH, BMP7, FGF8, FGF2, and insulin) can the brain organoids be further directed to a discrete brain region, such as cerebral cortex, optic cup, midbrain, hippocampus, thalamus, hypothalamus, cerebellum, ganglionic eminences, and choroid plexus [11–13, 15, 17, 30–38]. Importantly, brain region-specific organoids have been shown to have less variation across batches and cell lines, which makes experiments more reproducible and quantitative analyses more reliable and easier [18].

Nevertheless, the choice between unguided and guided methods should be dictated by the scientific questions of interest. Unguided brain organoids may be more ideal for questions related to spontaneous differentiation and self-organization properties during brain development, but at the price of having high variability and heterogeneity across samples. Guided brain region-specific organoids, in contrast, show less variability and heterogeneity and are thus best suited for questions related to cell fate specification, differentiation programs, and developmental trajectory within a specific brain region.

### 2.2. Recent Advances of Brain Organoid Techniques

**Figure 1:**
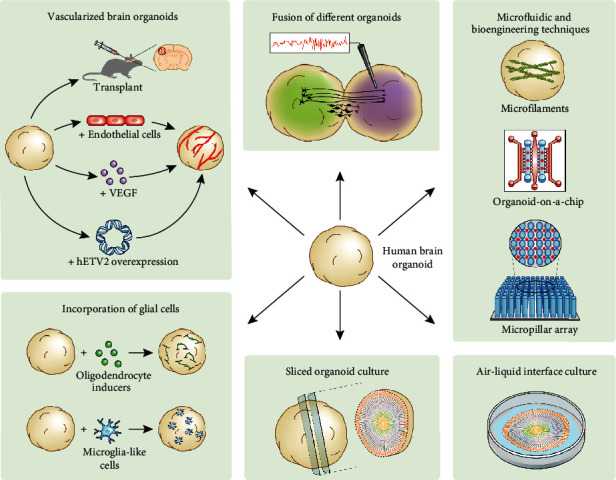
Recent advances of brain organoid techniques. (1) Different region-specific brain organoids can be fused together to generate so-called “assembloids” for the investigation of interregional interactions. (2) The lack of oligodendrocytes and microglia in cortical organoids has inspired the incorporation of these cell types into brain organoids. Strategies include exposure to oligodendrocyte inducers and coculturing with microglia-like cells. (3) The addition of vasculature in brain organoids is beneficial for oxygen and nutrient delivery under long-term culturing and hence the development of vascularized brain organoids. Strategies include transplantation of brain organoids into the mouse brain, coculturing with endothelial cells, exposure to vascular endothelial growth factor (VEGF), and overexpression of human ETS variant 2 (ETV2) in brain organoids. (4) Air-liquid interface culture technique has been shown to benefit neuronal survival and axonal growth. (5) Sliced organoid culture technique is able to overcome the diffusion limit in conventional brain organoid culture, leading to more expanded cortical plate and distinct layering of neurons. (6) Microfluidic and bioengineering techniques help improve the repeatability and uniformity of brain organoid culture, providing possibilities for generating organoids with simple procedure, high reproducibility, and low cost.

The tremendous promise of brain organoids in modeling human neurodevelopmental processes *in vitro* has inspired scientists to continuously innovate and improve the current methods. Recent advances include fusion of different brain region-specific organoids to model interactions between brain regions, incorporation of important cellular and structural components into brain organoids to better recapitulate features of the human brain, and other technical advances that benefit the development of brain organoids (Figure 1). We will review these major advances in this section.

#### 2.2.1. Fusion of Brain Region-Specific Organoids

Interregional interactions are critical processes in the developing brain. Although unguided brain organoids contain a variety of neural structures resembling interacting brain regions, they are less efficient to utilize in scientific experiments due to having high variability and heterogeneity among individuals. To improve the current methodology, brain region-specific organoids are generated separately as desired and fused together via coculture to form “assembloids,” by which developmental processes such as cellular interactions between distinct regions, synaptic formation, and establishment of early circuits can be investigated [39–42]. For example, the fusion of ventral and dorsal forebrain organoids revealed a unidirectional cell migration pattern; ventral-derived inhibitory neurons and interneurons were both observed to migrate in a saltatory pattern, with a single or branched process leading towards the dorsal side as previously reported in animal models [39–41]. These interneurons, after migrated into the dorsal side, exhibited increased branching complexity, showed changes in gene expression profiles, and connected and formed microcircuits with dorsal-derived excitatory neurons [40]. Similarly, corticothalamic interactions that are critical for sensory-motor processing were modeled by fusing cortical and thalamic organoids together [42]; corticostriatal circuits that regulate motivated behaviors and movements were also modeled by assembling human striatal spheroids with cortical organoids [43]. Notably, corticostriatal assembloids from patients with 22q13.3 deletion exhibited disease-associated defects in calcium activity [43], indicating the possibilities of using patient-derived assembloids in the investigation of disease-related interregional connectivity.

Most recently, a three-part system resembling the corticospinal-motor circuit was established by assembling human cortical spheroids, hindbrain/cervical spinal cord spheroids, and skeletal muscle spheroids together. Results have shown that stimulation of cortical spheroids triggered robust contraction of muscle spheroids, and these assembloids were able to stay intact both morphologically and functionally for up to 10 weeks postfusion [44], suggesting the possibilities of modeling more complex circuits with multipart assembloids. Despite the promising results found in these studies, further investigations are needed to examine whether assembloids actually model the endogenous regional interactions and, if so, what stage of development they are modeling.

#### 2.2.2. Incorporation of Glial Cells

Glial cells have fundamental roles in the regulation and support of the nervous system. Despite having astrocytes and oligodendrocyte progenitor cells developed in cortical organoids after long-term culturing [17, 18, 20, 40], mature oligodendrocytes have not been observed in typical cortical organoids [18, 45]. As oligodendrocytes are essential for many developmental processes, such as myelination, axonal maintenance, and nutrition and metabolic support of neurons, it is important to establish a system where they can be generated and functioning. By exposing cortical spheroids to certain differentiation inducers and accelerating such process with promyelinating drugs, oligodendrocyte-like cells are generated in “oligocortical spheroids” with features consistent to those of functionally mature oligodendrocytes [46]. Later on, a protocol that promotes the development of so-called human oligodendrocyte spheroids, which contains oligodendrocytes, astrocytes, and neurons, was established and thus provided a system to investigate oligodendrocyte development, myelination, and interactions with other cell types [47].

Another major subtype of glial cells is microglia, which act as the immune cells of the nervous system and regulate its health by responding to inflammation, phagocytosing infectious microorganisms, and pruning redundant synapses. However, despite being innately developed within unguided and self-organized cerebral organoids [48], microglia are completely absent from guided cortical organoids as they originate from nonneural lineage. Dysregulation of microglia has been shown to affect normal brain function and contribute to neurodegenerative disorders such as Alzheimer's and Parkinson's disease [49–51], and hence the importance of establishing microglia-containing brain organoids. Attempts have been made by coculturing microglia-like cells with neuron aggregates or brain region-specific organoids [52–55]. Notably, microglia migrated into the organoid would cluster near an injured site and change morphology to that of activated microglia upon injury of the central nervous system*s* [52]. Moreover, differential cellular phenotypes were observed between the coculture of microglia-like cells with dorsal organoids and with ventral organoids, including differences in migration ability, intracellular Ca^2+^ signaling, and the response to proinflammatory stimuli [55]. Changes of gene expression in microglia-like cells before and after coculturing were detected by transcriptome analysis [53–55], prompting interests in studying how the presence of microglia in brain region-specific organoids will in return affect their development and functions.

#### 2.2.3. Incorporation of Structural Components

Due to being derived from nonneural lineage, functional vasculature is absent in brain organoids, resulting in the insufficient delivery of oxygen and nutrient into organoids under long-term culturing and hence the increased apoptosis and cell death in the inner zone that forms a necrotic core [56–58]. Functional vasculature is critical for the differentiation and maturation of neuronal/glial progenitor cells [59], and thus, several approaches have been established in attempts to induce vascularization of brain organoids. Coculturing of cerebral organoids at early developmental stage with endothelial cells allowed robust vascularization of the organoid after 3-5 weeks *in vitro* or 2 weeks *in vivo* after transplanted into immunodeficient mice, in which human CD31^+^ blood vessels were found inside and in-between rosettes within the center of the transplanted organoid [60]. Other approaches, including induction of endothelial cell differentiation in cerebral organoids by vascular endothelial growth factor (VEGF) treatment [61] or by overexpressing human ETS variant 2 (ETV2) [62], as well as coculture of hPSCs with human umbilical vein endothelial cells [63], have also successfully generated a functional vascular-like system in brain organoids without affecting neurogenesis. More importantly, vascularized organoids acquired many characteristics of blood-brain barrier, including expression of tight junctions, molecular transporters, and other genes related to blood vessel morphogenesis, and supported the formation of blood vessels *in vivo* [61–63], providing a potential platform for studying blood-brain barrier and drug discovery.

#### 2.2.4. Other Technical Advances

Additional advances mainly focus on the improvement of organoid culture system, either by alternative culture techniques that allow better recapitulation of neurogenesis or by state-of-art bioengineering technologies that increase the repeatability and uniformity of brain organoid cultures. For example, air-liquid interface culture techniques were established to improve neuronal survival and axonal growth, resulting in active neuronal networks and circuit formation with functional neuronal output [64]. Later on, a sliced neocortical organoid system was established, which overcame the diffusion limit in typical brain organoids and prevented cell death over long-term culturing. Sustained neurogenesis, which led to an expanded cortical plate, was observed by this system, forming distinct upper and deep cortical layers for neurons and astrocytes similar to the neocortex in the third trimester [65].

Additionally, the application of state-of-art microfluidic and bioengineering techniques has greatly improved the repeatability and uniformity of brain organoid culture. For example, poly (lactide-co-glycolide) copolymer (PLGA) fiber microfilaments were engineered to be used as a floating scaffold to generate elongated embryoid bodies, which then self-organized into cerebral organoids, with more-consistent formation of enlarged ventricular structures and neuroepithelium [66]. Moreover, microchip culture systems have been developed and utilized to generate brain organoids in confined compartments for the investigation of surface wrinkling, a biological process that is significant for the formation of gyrus and sulcus formation in the cortical plate. In this study, two opposing forces, the cytoskeletal contraction at the organoid core and the nuclear expansion during cell cycle at the organoid perimeter, were identified contributing to the formation of surface wrinkling [67]. More recently, benefited from the rapid development of microfluidic devices and the establishment of air-liquid interface culture techniques, a one-stop microfluidic platform has been developed to generate and culture cerebral organoids for investigating the effect of prenatal cannabis exposure on early brain development [68]. This platform is advantageous in that it greatly simplifies the experimental procedure and improves productivity. Hopefully with the continuous advances and improvement of culture techniques and bioengineering technology, brain organoid can soon become a sophisticated model system that not only recapitulates human brain development but also has the characteristics of fast generation, high reproducibility, and low cost.

### 2.3. Disease Modeling Using Brain Organoids

Brain organoids, owing to having 3D structures mimicking key features of the developing brain, are particularly suitable for translational research. Patient iPSC-derived brain organoids, for instance, contain genetic abnormalities that lead to the disease and are therefore capable of recapitulating the disease pathology as well as phenotypes in a dish. On the other hand, isogenic brain organoids generated via gene-editing techniques can help reveal the necessity and essentiality of a specific gene mutation to the disease. As a result, brain organoids have been extensively explored for the modeling of various neurological disorders, including neurodevelopmental disorders, neurodegenerative disorders, infectious diseases, and brain cancers. We will summarize and discuss some of these studies in this section (Table 1).

#### 2.3.1. Modeling Neurodevelopmental Disorders

*(1) Primary Microcephaly*. Primary microcephaly, also known as autosomal recessive primary microcephaly (MCPH), is a condition where abnormalities occur at the early developmental stage of the human brain, resulting in reduced head circumference and most likely intellectual disability and seizures [69]. Well-known genetic causes of primary microcephaly are mainly genes involved in the assembly of centrosomes and cilium, such as *CDK5RAP2*, *ASPM*, *CPAP*, and *WDR62* [14, 45, 70–72]. However, rodent models of primary microcephaly did not exhibit a significantly reduced brain size as observed in human [73, 74], and thus prompting interest in developing human-specific models of this disease.

The first microcephalic cerebral organoids were derived from iPSCs of a microcephaly patient, harboring heterozygous truncation mutations in CDK5 regulatory subunit-associated protein 2 (CDK5RAP2), a component of the pericentriolar material (PCM) in centrosomes that regulates the organization of spindle microtubules [14]. The mutant organoids were significantly smaller in size and exhibited reduced number of progenitor cells as well as premature neuronal differentiation compared to the controls. RNAi-mediated knockdown of *CDK5RAP2* in the control organoids recapitulated the mutant phenotypes, while overexpression of this gene in the mutants rescue the phenotypes [14]. Later on, patient iPSC-derived cerebral organoids harboring mutations in the abnormal spindle-like microcephaly-associated (*ASPM*) gene were generated [70]. ASPM is a mitotic spindle protein; mutations in the *ASPM* gene are the most common cause for primary microcephaly. These mutant organoids exhibited significantly reduced overall size, fewer progenitor cells in both ventricular zone and outer subventricular zone, poor lamination, and a reduction in neurons with calcium activity [70]. Centrosomal-P4.1-associated protein (CPAP) is a centriole wall protein required for the assembly and recruitment of PCM proteins to the centrosome; mutations in the *CPAP* gene can cause Seckel syndrome and microcephaly. Brain organoids generated from the iPSCs of a Seckel syndrome patient were significantly smaller in size [75]; NPCs in these mutant organoids had delayed cilia disassembly that caused a retardation in cell cycle progression, leading to premature differentiation of NPCs into early neurons and thus an overall reduction in the progenitor pools [75]. Similarly, *WDR62* ablated iPSC-derived brain organoids showed delayed cilia disassembly and retarded cell cycle progression, resulting in reduced proliferation and premature differentiation of NPCs [71]. It turns out that WDR62 interacts with CEP170, promoting CEP170 to locate in the matrix of primary cilia; CEP170 then recruits the microtubule depolymerization factor KIF2A to disassemble cilium [71].

*(2) Autism Spectrum Disorder*. Autism spectrum disorder (ASD) is a developmental condition related to neurodevelopment that affects a person's perception and interaction with other people, characterized by difficulties in communication and social-emotional reciprocity, restricted interests, and repetitive behavior. The utilization of brain organoids has deepened our understanding on the cellular and molecular mechanisms of ASD pathophysiology. Cortical organoids generated from the iPSCs of severe idiopathic ASD patients exhibited upregulation of genes involved in cell proliferation, neuronal differentiation, and synaptic assembly, as well as cellular alterations including accelerated cell cycles and increased number of GABAergic neurons [15]. *FOXG1* was one of the most upregulated genes in ASD organoids; RNAi-mediated knockdown of *FOXG1* was able to rescue the overproduction of GABAergic neurons, suggesting that the overexpression of *FOXG1* may initiate a shift towards the GABAergic lineage, which results in an imbalance between excitatory and inhibitory neurons and eventually leads to ASD [15]. In addition to *FOXG1*, an exome-sequencing study has identified *CHD8* (chromodomain helicase DNA-binding protein 8) as one of the most commonly mutated genes in ASD. Combined with the CRISPR/Cas9 gene-editing technique, cerebral organoids harboring a heterozygote mutation of *CHD8* (*CHD8*^+/-^) were generated [76]. Differentially expressed genes (DEGs) between heterozygote mutant organoids and isogenic controls were identified by RNA-sequencing; pathway analysis revealed an upregulation of genes involved in neurogenesis, neuronal differentiation, forebrain development, Wnt/*β*-catenin signaling, and axonal guidance [76]. This study, again, highlights the possibility that the imbalance between excitation and inhibition in the brain is a pathogenic cause of ASD.

*(3) Tuberous Sclerosis Complex*. Tuberous sclerosis complex (TSC) is an autosomal dominant genetic disorder characterized by the growth of benign tumors in multiple organ systems including the brain, kidneys, lungs, and skin. Among these manifestations, neurological abnormalities attract the most attention due to being the most complicated and therapeutically challenging conditions in TSC. In addition to brain lesions such as cortical tubers (focal regions of disorganized and dysmorphic neurons and glia), subependymal nodules, and subependymal giant cell astrocytomas, neurological deficits such as epilepsy, ASD, and intellectual disability are often seen in TSC patients [77–79]. Studies have shown that mutations in the *TSC1* or *TSC2* gene are the causes of TSC as they lead to *TSC1/TSC2* deficiency in organs and hyperactivation of the mTOR signaling pathway, which plays an important role in regulating cell growth and proliferation [79, 80]. So far, the molecular mechanisms underlying TSC are still unclear. A recent study using CRISPR/Cas9-mutated *TSC1* and *TSC2* cortical spheroids revealed that homozygous knockout of *TSC1* or *TSC2* disrupted the developmental suppression of mTORC1 signaling, resulting in reduced neurogenesis, increased gliogenesis, and dysmorphia of neurons and glia similar to those observed in patients' cortical tubers [81]. Moreover, it has been found that biallelic inactivation of *TSC2* was necessary and sufficient to cause the formation of dysplastic cells in cortical spheroids. Therapeutically, it has been shown that treatments with rapamycin since either early stage (day 12-110) or later stage (day 80-110) of development strongly reduced mTORC1 signaling and reversed cellular hypertrophy in *TSC2*-deficient spheroids. However, only early treatment with rapamycin could rescue neuronal differentiation defects in *TSC2*-deficient spheroids, and continuous treatments were required to sustain these effects, highlighting the importance of timing and duration of pharmacological treatments [81].

#### 2.3.2. Modeling Congenital/Infectious Diseases

*(1) Neonatal Hypoxia-Ischemia Injury*. Neonatal hypoxic-ischemia (HI) injury, synonymous with hypoxic-ischemic encephalopathy (HIE) that occurs at 36 gestational weeks or later, is the most common cause of death and disability in neonates. Even though early interventions and improvements in care have led to an increase in survival rate after hypoxic insult, many survivors still suffer from life-long neurodevelopmental deficits such as cerebral palsy, seizures, epilepsy, and cognitive impairment [82, 83]. Recently, in order to better examine the effects of hypoxia on neurodevelopment, cerebral organoids of neonatal HI were generated and cultured at different oxygen concentrations [82]. Hypoxic environment had an inhibition effect on dorsal-related genes such as *FOXG1*, *CTIP2*, and *TBR1* but had no effect or minimal effect on more ventral genes such as *ENG1*, *DLX2*, and *NKX2.1*. Notably, the inhibition of dorsal genes under hypoxic environment could be alleviated by the application of minocycline, demonstrating the therapeutic potential of this small molecule [82]. Another study using hiPSC-derived 3D-cultured cortical spheroid revealed a reduction of TBR2^+^ intermediate progenitors after 48-hour cultivation under hypoxic environment [84]. This cell-specific defect was related to changes in the unfolded protein response (UPR) pathway in TBR2^+^ progenitors, resulting in cell cycle damage and premature neural differentiation in these cells. Treatments with the UPR modulator ISRIB were able to rescue these phenotypes observed after the hypoxic insult [84].

*(2) ZIKV Infection*. In addition to the well-known genetic causes mentioned in the previous section, external factors such as viral infection and environmental cues can also lead to microcephaly, which is termed acquired microcephaly. Zika virus (ZIKV) is a member of the flavivirus family. Zika virus (ZIKV) infection is the most studied condition as its outbreak in South America cooccurred with an increased incidence of microcephalic neonates, arousing suspicion in a causal relationship between the two. Due to the inaccessibility of live infected human fetal tissues and the variability of postmortem tissues, brain organoids have been widely used to model ZIKV infection and investigate the cellular mechanisms underlying it. For example, hiPSC-derived forebrain organoids exposed to ZIKV revealed specific tropism of ZIKV towards NPCs over intermediate progenitor cells or immature neurons in the organoids [17]. Infected NPCs provided material and machinery for virus production, leading to the amplification of ZIKV and the propagation of infected cells over time [17, 85]. Transient exposure (i.e., one day) of early-stage forebrain organoids to ZIKV was sufficient to cause microcephalic-like phenotypes, including thinning of the neuronal layer, decrease in overall size, and dilation of the ventricular lumen, which was in agreement with the clinical finding that ZIKV infection during the first trimester is the most dangerous [17]. Mechanistically, it has been shown that suppression of NPC proliferation and increased cell death in ZIKV-infected forebrain organoids were responsible for the decrease in organoid size [17]. Remarkably, these effects of ZIKV infection are not a general feature of viruses in the flavivirus family as exposure of cerebral organoids to dengue virus 2 (DENV2), another member in the flavivirus family that causes dengue fever, did not attenuate NPC growth [85]. Meanwhile, different strains of ZIKV were tested to see if there is intrinsic difference in the pathogenicity of virus. Interestingly, ZIKV^B^, a more recent clinical isolate from Brazil, appeared to have stronger deleterious effects in cerebral organoids than the original African strain ZIKV^M^, showing more severe NPC depletion and neuronal layer disruption [86]. However, it is worth noting that passage history is important for the pathogenicity of virus and thus should be taken into consideration when drawing conclusions.

Other studies focused on the molecular mechanisms of ZIKV infection have revealed several biological pathways affected by the virus. For example, transcriptome analysis of human cerebral organoids infected with ZIKV exhibited upregulation of toll-like receptor 3 (TLR3), an innate immune receptor [87]. Further analysis revealed a TLR3-mediated downregulation of neurogenesis and upregulation of proapoptotic pathways in the infected organoids. Interestingly, a direct competitive TLR3 inhibitor rescued ZIKV-mediated apoptosis and partially rescued the reduced size of infected organoids [87]. Later on, another study also revealed activated innate immune responses in ZIKV-infected cortical organoids, which could explain the increased progenitor apoptosis and restricted growth of infected organoids [88]. Interestingly, administration of either duramycin or ivermectin to infected organoids dramatically reduced the teratogenic effects of ZIKV infection on cortical development, highlighting the potential therapeutic role of these drugs in anti-ZIKV infection [88]. Translational studies have also been performed to search for potential therapeutic agents that could alleviate ZIKV-mediated phenotypes. A high-content screening in hiPSC-derived NPCs identified hippeastrine hydrobromide (HH) and amodiaquine dihydrochloride dihydrate (AQ) as drug candidates to inhibit ZIKV infection [89]. It has been shown that HH rescued ZIKV-mediated growth and differentiation defects in NPCs and was even capable to suppress viral propagation in adult mice with active ZIKV infection [89]. Additionally, a recent study revealed an abundant production of virus-induced small interfering RNAs (siRNAs) in NPCs [90]. Ablation of key components in RNAi machinery significantly enhanced ZIKV replication in infected cells, and thus prompting interest in testing the effects of RNAi enhancers on these cells. Remarkably, enoxacin, an RNAi enhancer, completely prevents ZIKV infection and rescued ZIKV-mediated microcephalic-like phenotypes in infected organoids [90], bringing RNAi into the discussion of potential therapeutic targets.

*(3) SARS-CoV-2 Infection*. Severe acute respiratory syndrome coronavirus 2 (SARS-CoV-2) infection has caused the COVID-19 global pandemic since 2019, resulting in more than 216 million infected people and over 4.5 million deaths worldwide as of August 2021 (https://covid19.who.int). Even though the infection primarily affects the respiratory system, neurological complications have been reported in a significant number of patients, including headache, dizziness, cerebrovascular injury, encephalitis, hypogeusia, and hyposmia, as well as neuropsychiatric symptoms such as confusion and new-onset psychosis [91–94]. Although a few cases reported the presence of viral RNA in the brain and cerebrospinal fluid (CSF) of infected patients [93, 95–97], it is hard to draw conclusions on the prevalence of central nervous system infection based on these sporadic reports. Therefore, it remains unclear whether the neurological symptoms in COVID-19 are caused by direct neural infection or by some more indirect mechanisms. Due to the difficulties in accessing human brain tissue, brain organoids were utilized to investigate this question. By exposing hiPSC-derived monolayer cortical neurons, astrocytes, and microglia, as well as 3D-cultured cortical, hippocampal, hypothalamic, and midbrain organoids to SARS-CoV-2, the viral tropism in various cell types was revealed [98]. It has been shown that SARS-CoV-2 had limited tropism for neurons and astrocytes under clinically relevant conditions but rather had a particularly high rate of infection in choroid plexus (ChP) epithelial cells, a cell type present in some of the hippocampal organoids tested in this study [98, 99]. Indeed, this finding was confirmed in further examinations using choroid plexus organoids (CPOs), from which a productive infection of SARS-CoV-2 in ChP epithelial cells was revealed [98, 100]. This high susceptibility of CPOs to SARS-CoV-2 may be explained by the finding that ACE2 and TMPRSS2, the key cell entry receptors for SARS-CoV-2, were highly expressed in the ChP *in vivo* and *in vitro* [98, 100, 101]. The infection of SARS-CoV-2 in CPOs caused an increase in both cell-autonomous and non-cell-autonomous cell death, transcriptional dysregulation, and disruption of ChP epithelial integrity and barrier function [98, 100]. In fact, recent clinical data reported leakage of blood proteins into CSF in more than 40% of patients tested [97], which was in support of this finding as the disruption of ChP integrity would be expected to lead to leakage in the blood-CSF barrier (B-CSF-B). Subsequently, a breakdown of the B-CSF-B would allow abnormal entry of immune cells and cytokines, which could lead to harmful neuroinflammation and neural tissue injury. Taken together, so far, it has been proposed that the neurological symptoms in COVID-19 patients are more likely to be consequences of indirect effects of viral infection. However, this proposal requires further verifications by animal models and postmortem ChP from infected patients, as current clinical data did not reveal high prevalence of SARS-CoV-2 in the circulating bloodstream [96], raising questions on the pathway(s) of viral entry.

On the other hand, the remarkable variability in terms of symptom severity among infected individuals has prompted interest in investigating the potential molecular mechanism(s) underlying it. A recent study reported the host gene FURIN as a mediator for SARS-CoV-2 infection and a common variant rs4702 that is located in the 3′UTR of this gene being an influencer of SARS-CoV-2 infection *in vitro*. Moreover, CRISPR/Cas9-mediated allelic conversion (from AA to GG) at rs4702 decreased the neuronal and alveolar expression of FURIN and led to reduced SARS-CoV-2 infection [99], which was in agreement with the idea that host genome is associated with SARS-CoV-2 infection and might dictate the severity of clinical outcomes.

#### 2.3.3. Modeling Neurodegenerative Disorders

*(1) Alzheimer's Disease*. Alzheimer's disease (AD) is the most common neurodegenerative disease and is characterized by progressive decline in memory, thinking, language, behavior, and other cognitive abilities. On a cellular level, AD is characterized by the extracellular deposition of *β*-amyloid plaques as well as intracellular formation of neurofibrillary tangles that are composed of aggregated hyperphosphorylated tau (pTau). Even though brain organoids are thought to recapitulate embryonic brain development, which seems far from neurodegeneration, several studies have reported successful establishment of brain organoids harboring AD-like pathologies. For example, an early study revealed that 3D-differentiated neuronal cells overexpressing APP or PSEN1 gene variants from familial AD (fAD) patients exhibited robust deposition of *β*-amyloid plaques and aggregates of pTau, recapitulating the two pathological hallmarks of AD. Similarly, AD-like pathologies were observed in fAD patient iPSC-derived brain organoids, including *β*-amyloid (A*β*) aggregation, hyperphosphorylated tau, and endosome abnormalities. These pathologies were excluded from various control lines and occurred at consistent incidence among several fAD lines that carried different mutations [102, 103]. Moreover, treatments with *β*- and *γ*-secretase inhibitors were able to significantly reduce amyloid and tau pathology in AD-like brain organoids [103, 104], suggesting the potential of utilizing these organoids as platforms for preclinical drug discovery in AD.

Other studies focus on the investigation of sporadic AD (sAD). *APOE4* is the E4 allele of *APOE* and is the earliest identified and most significantly associated genetic risk factor for sAD, leading to increased AD risk relative to the *APOE3* allele [105–108]. Isogenic *APOE4* brain organoids, which were generated by switching the *APOE3* allele in healthy individual iPSCs to *APOE4* allele via CRISPR/Cas9 gene-editing technique, showed an increased A*β* accumulation and pTau compared to *APOE3* organoids. Conversely, switching *APOE4* in sAD patient iPSCs to *APOE3* was sufficient to alleviate most of the AD-related phenotypes in brain organoids, supporting the central role of *APOE4* in sAD pathology [109].

*(2) Parkinson's Disease*. Parkinson's disease (PD), being the second most common neurodegenerative disease after AD, is a chronic and progressive nervous system disorder affecting movement. Symptoms commonly include tremors, slowness in movement, muscle stiffness, and difficulties with speech, balance, and coordination. On a cellular level, PD is characterized by the loss of dopaminergic neurons in the substantia nigra of the midbrain as well as the development of neuronal Lewy bodies (*α*-synuclein) [110, 111]. The current cellular and animal models have some limitations in recapitulating pathological hallmarks of PD [112], leading to the development of midbrain organoids (MOs) as a better alternative for modeling PD *in vitro* [113, 114]. Previous studies have shown that missense mutations in the leucine-rich repeat kinase 2 (*LRRK2*) gene locus, particularly *LRRK2* G2019S mutation, are common causes of late-onset familial and sporadic PD [115, 116], prompting interest in studying the pathogenic mechanisms of LRRK2-associated PD. In a recent study, isogenic MOs harboring a *LRRK2* G2019S mutation were generated from CRISPR/Cas9-edited iPSCs. These organoids exhibited several PD-like phenotypes, including shortened neurite length in dopaminergic neurons (mDANs), decreased expression of mDAN-specific marker (e.g., TH, AADC, and DAT), and increased aggregation and abnormal clearance of *α*-synuclein. Notably, analysis of differentially expressed genes revealed an upregulation of TXNIP, a thiol-oxidoreductase, in the *LRRK2*-G2019S mutant organoids specifically; inhibition of this gene was able to ameliorate the mutant phenotypes induced by *LRRK2*-G2019S mutation, indicating the possibility of TXNIP in mediating disease phenotypes of patients with *LRRK2*-associated PD [113]. In line with these findings, another study using MOs derived from PD patients who carried the *LRRK2* G2019S mutation also demonstrated a decrease in the number and complexity of mDANs compared to the control organoids [117]. Moreover, FOXA2-positive progenitor cells were found to be significantly increased in these patient-derived organoids, suggesting a neurodevelopmental defect is likely associated with the *LRRK2* G2019S mutation. Importantly, it has been shown that introduction of the LRRK2-G2019S mutation within a healthy background was sufficient to cause deleterious effects on the complexity of mDANs, consistent with the findings in Kim et al. (2019), and yet, correction of the LRRK2-G2019S mutation within a PD patient background was not able to rescue the mutant phenotypes [117], supporting the hypothesis that genetic background of PD patients may influence the *LRRK2*-induced mDAN degeneration [118].

Additionally, as homozygous loss-of-function mutations in DNAJC6 were previously identified in familial juvenile/early-onset PD [119–121], MOs harboring CRISPR/Cas9-mediated DNAJC6 mutations were generated and utilized for investigating the roles of DNAJC6 in PD pathogenesis [114]. These mutant organoids exhibited key PD pathologic features, including mDAN degeneration, *α*-synuclein aggregation, increased neuronal firing frequencies, and mitochondrial and lysosomal defects. DNAJC6 ablation also led to impairment of WNT-LMX1A regulation, which is critical for early ventral midbrain (VM) patterning and mDAN development, and thus resulted in VM patterning defects and vulnerable mDANs in mutant MOs [114]. Moreover, MOs derived from idiopathic PD patients were also utilized for investigating the pathophysiology of this disease subtype [122]. Changes in the expression of LIM homeobox transcription factor alpha (early) and tyrosine hydroxylase (late) markers were observed in patient-derived MOs; several crucial genes associated with idiopathic PD, e.g., TH, PTX3, LMX1A, and FOXA2, were also identified in this study [122].

*(3) Huntington's Disease*. Huntington's disease (HD) is an autosomal dominant genetic disorder characterized by motor impairments such as chorea, dystonia, and incoordination, cognitive decline such as forgetfulness, impaired judgement, and learning difficulties, and psychiatric problems such as insomnia and depression. The cause of HD has been shown to be a polymorphic CAG repeat expansion in the *huntingtin* (*HTT*) gene located on chromosome 4 that leads to abnormal degeneration of neurons within the striatum and cortex [123, 124] through several biological mechanisms including altered gene expression profile, disrupted mitochondrial and metabolic function, direct toxicity of the mutant protein, and aberrated ATP levels. Also, the length of CAG repeats in the *HTT* gene has been found to be crucial for disease onset and severity: fewer than 36 repeats are normal; 36–39 repeats are abnormal but might result in HD with reduced penetrance; more than 40 repeats result in adult-onset HD; and more than 60 repeats generally result in Juvenile Onset HD (JHD) [125, 126]. As JHD progresses significantly faster than adult-onset HD, researchers have brought up the possibility that mutant HTT may lead to neurodevelopmental deficits in addition to neurodegenerative manifestations in HD. Indeed, many studies have examined the role of HTT in brain development in both rodent models and monolayer cell cultures [127–132], and yet, the impact of mutant HTT on neurodevelopment, especially early neurogenesis and cortical layer formation, was less clear, most likely due to the difficulties of accessing human embryonic brain tissues with HD. To address this question, one group took advantage of patient iPSC-derived cerebral organoids to investigate early neurodevelopmental processes in HD [133]. They found that CAG repeat expansion caused significant defects in early telencephalic induction and progenitor identity acquisition, leading to abnormal neuronal specification and disrupted cellular organization. They also observed severer phenotypes in the organoids with larger repeat expansion than those with shorter expansion, which were in line with the clinical representation that the longer the CAG repeats are, the earlier and severer the symptoms tend to manifest [133]. A later study using cerebral organoids derived from patient iPSCs and a panel of TALEN-mediated isogenic HD hESCs reported similar results, as HD organoids showed impaired cell cycle regulatory processes and reduced symmetric division of apical progenitors that eventually led to disrupted neuroepithelial structures and premature neurogenesis in these organoids [134].

#### 2.3.4. Modeling Brain Cancer

Glioblastoma (GBM) is the most lethal and devastating type of glioma, accounting for 54% of all gliomas [135]. Current treatments are very limited and mainly focus on slowing the progression of the cancer and reducing signs and symptoms, as the rapid development and invasion of GBM often make surgical resection improbable. The prognosis of GBM is dismal, with a median survival time of approximately 15 months and a 5-year survival rate of less than 5% [136]. In order to study the formation and progression of GBM *in vitro*, several strategies have been taken. For example, unguided cerebral organoids were utilized, in which oncogenes and/or tumor suppressors were manipulated using CRISPR/Cas9- and/or transposon-mediated approaches to induce mutagenesis and tumorigenesis [137, 138]. Many features of GBM cells were observed in transformed organoids, including capability of expansion and invasion (both *in vivo* and *in vitro*), cellular markers, and gene expression profiles [137, 138]. Another strategy was taken by coculturing either patient-derived glioma stem cells (GSCs) [139] or glioblastoma spheroid [140] with human cerebral organoids. Both studies revealed a rapid and deep invasion of glioblastoma cells into the host tissue, forming hybrid organoids that exhibited an invasive tumor phenotype [139, 140]. Such GBM hybrid organoids would provide a scalable and easily manipulable system for the investigation of tumorigenesis and progression, as well as for the screening of anticancer drugs [141, 142]. More recently, a different method was established, generating glioblastoma organoids (GBOs) directly from resected tumor tissue without additional manipulation [143]. These GBOs recapitulated inter- and intratumoral heterogeneity as well as many key features of glioblastoma, including histological features, cell type diversity, transcriptomic signatures, mutation profiles, and aggressive infiltration after transplantation. This method allows for rapid generation of patient-specific glioblastoma organoids, which can be utilized for testing personalized therapies, treatments, and drugs [143].

## 3. Conclusions and Discussion

With less than a decade of development, brain organoid technology has revolutionized our toolbox for investigating cellular and molecular mechanisms of neurodevelopment and neural disorders. In this review, we summarized many recent advanced techniques in the field of brain organoids, such as the development of assembloids, incorporation of cellular and structural components, and other optimized culture systems. We also discussed some of the translational applications of brain organoids, including disease modeling and screening or testing potential pharmaceutical compounds. Attracted by the unique advantages of brain organoids, more and more researchers devoted themselves into this field and established many more disease models for the investigation of disease mechanisms. For example, most recently, MECP2 knockout neurospheres and cortical organoids were generated for modeling Rett syndrome [144]; Down syndrome cerebral organoid models were established from patient-derived iPSCs [145]; and iPSC-derived brain organoids infected by a “clinical-like” human cytomegalovirus (HCMV) strain were utilized for studying HCMV-induced microcephaly [146]. Furthermore, such disease-modeling organoids also provide a platform for drug screening [88, 89] and act as a subject in the investigation of potential organoid transplantation therapy for neurological disorders [147–149].

Despite the numerous promising results researchers have obtained from brain organoid models, there are still limitations in the current system. Firstly, as NPCs with high metabolic demands are often located in the inner zone of brain organoids, continuous apoptosis and cell death caused by the insufficient delivery of oxygen and nutrients to the inner zone have greatly hampered the neurogenesis and further maturation of brain organoids, leading to the incompetence of modeling late-stage events such as distinct cortical layering, cortical expansion, and cortical folding. Improvements can be achieved by overcoming the diffusion limit in long-term organoid cultures. For example, the use of spinning bioreactors or orbital shakers as well as elevated oxygen concentration in the incubator has been shown to be beneficial in some ways [13, 17, 66]. Alternative culture methods such as an air-liquid interface culture system [64] and sliced neocortical organoid system [65] have also contributed to the development of a better organoid model. Additionally, there is no doubt that the incorporation of vasculature into brain organoids would largely improve the delivery of oxygen and nutrients. Methods involving building or providing a vascular system in brain organoids, such as constructing vascular-like networks with perfusion via bioengineering or grafting organoids into animal brains to allow invasion of the host vasculature, are therefore being actively studied and developed [150].

Secondly, a recent study has revealed that brain organoids generated from current methods did not resemble their cortical progenitor counterparts at the earliest developmental stages, despite having increased fidelity of cell types after the radial glia and neuronal populations emerged [151]. Specifically, a mesenchymal-like population marked by ALX1 and LUM expression was identified in samples at or before Carnegie stages (CS) 16 but was not detected in cortical organoids until week 7, highlighting the importance of continuing optimizing brain organoid protocols for the investigation of developmental processes prior to neurogenesis [151].

Another major limitation of the current organoid model is that the maturation process takes too long and is therefore costly and labor-intensive. Future improvements in terms of speeding up this process would not only benefit the generation of brain organoids but also create a more “aged” model for studying age-dependent neurodegenerative disorders. Moreover, the use of bioengineering technology such as microfluidics, biomaterial, and bioprinting may further improve the efficiency of generating organoids with low variability, high reproducibility, and low cost.

Lastly, the introduction of assembloid has opened a new avenue for the investigation of interregional connections and activities using different guided brain region-specific organoids. Future directions include establishing more sophisticated assembloid systems that compose more brain regions as well as incorporating nonneural lineages such as microglia, endothelial cells, hematopoietic cells, and meninges into the assembloid to better mimic the *in vivo* condition. The ultimate goal is to assemble a whole brain-like structure that comprehensively models human brain development and function.

Taken together, brain organoid technology, although still being at its primary stage, has become an invaluable tool for studying neurodevelopment and neural disorders. While new methods and improvements are being made to generate more advanced organoid systems, it is important to keep in mind that no model is perfect. Thus, we should always choose a model system based on the biological question of interest and be cautious when drawing conclusions. Only when interpreted comprehensively and complementarily with other models can we gain new insight into the biological basis of human brain development.

## Figures and Tables

**Table 1:** Selected studies investigating neurological disorders/deficits using human brain organoids.

Disease	Studies	Organoid type	Methods of generation	outcomes
Primary microcephaly	Lancaster et al., 2013 [14]	Cerebral organoids	Patient iPSC-derived; *CDK5RAP2* mutation	Fewer progenitor cells, premature neuronal differentiation; *CDK5RAP2* overexpression rescued the mutant phenotypes
	Li et al., 2017 [70]	Cerebral organoids	Patient iPSC-derived; *ASPM* mutation	Reduced organoid size, fewer progenitor cells in VZ and oSVZ, poor lamination, reduced neuronal calcium activity
	Gabriel et al., 2016 [75]	Cerebral organoids	Seckel patient iPSC-derived; *CPAP* mutation	Delayed cilia disassembly led to premature differentiation of NPCs and reduced progenitor pools
	Zhang et al., 2019 [134]	Cerebral organoids	hPSC-derived; CRISPR/Cas9-mediated homozygous knockout of *WDR62*	Delayed cilia disassembly and retarded cell cycle progression led to reduced proliferation and premature differentiation of NPCs

Autism spectrum disorder (ASD)	Mariani et al., 2015 [15]	Cortical organoids	Idiopathic ASD patient iPSC-derived	Altered transcriptomic profiles, particularly *FOXG1* upregulation; accelerated cell cycles; increased GABAergic neuron production, can be rescued by RNAi-mediated *FOXG1* knockdown
	Wang et al., 2017 [76]	Cerebral organoids	hiPSC-derived, CRISPR/Cas9-mediated heterozygous mutation of *CHD8* (*CHD8*^+/-^)	Upregulation of genes involved in neurogenesis, neuronal differentiation, forebrain development, Wnt/*β*-catenin signaling, and axonal guidance

Tuberous sclerosis complex (TSC)	Blair et al., 2018 [81]	Cortical spheroids	CRISPR/Cas9-mediated homozygous knockout of *TSC1* or *TSC2* in hESCs	mTORC1 hyperactivation, reduced neurogenesis, increased gliogenesis; dysplastic cells in *TSC2*^−/−^ cortical spheroids can be rescued by early and continuous rapamycin treatments

Neonatal hypoxia-ischemia injury	Boisvert et al., 2019 [82]	Cerebral organoids	hESC-derived; 72-hour under hypoxic environment	Inhibition of dorsal-related genes such as FOXG1, CTIP2, and TBR1; could be alleviated by minocycline
	Pasca et al., 2019 [84]	Cortical spheroids	hiPSC-derived; 48-hour under hypoxic environment	Reduction of TBR2^+^ intermediate progenitors led to cell cycle damage and premature neural differentiation; rescued by ISRIB treatments

ZIKV-associated microcephaly	Qian et al., 2016 [17]	Cortical organoids	hiPSC-derived; MR766 and FSS13025 ZIKV strain infected	Reduced organoid size, reduced neuronal layer thickness, expanded ventricular lumen, increased cell death
	Dang et al., 2016 [87]	Cerebral organoids	hESC-derived; MR766 ZIKV strain infected	Reduced organoid size, TLR3 upregulation and TLR3-mediated transcriptomic alterations; direct inhibition of TLR3 reduced phenotypes
	Watanabe et al., 2017 [88]	Cortical organoids	hPSC-derived; PRVABC59 ZIKV strain infected	Activated innate immune responses led to increased progenitor apoptosis and reduced organoid size; duramycin or ivermectin rescued the teratogenic effects of ZIKV infection

SARS-CoV-2-associated neurological deficits	Jacob et al., 2020a [98]	Cortical, hippocampal, hypothalamic, midbrain, and ChP organoids	hiPSC-derived; SARS-CoV-2 USA-WA1/2020 infected	Particular tropism for ChP epithelial cells, caused increased cell death, transcriptional dysregulation, disrupted ChP epithelial integrity and barrier function
	Pellegrini et al., 2020 [100]	Cerebral and ChP organoids	hPSC-derived; SARS-CoV-2 spike pseudovirus and live virus infected	Particular tropism for ChP epithelial cells of cerebral organoids; infected cells expressing ACE2 and lipoproteins; ChP epithelial integrity and barrier function were disrupted

Alzheimer's disease (AD)	Gonzalez et al., 2018 [102]	Cerebral organoids	Familial AD or DS patient iPSC-derived	*β*-Amyloid (A*β*) aggregation, formation of neurofibrillary tangle-like structures, hyperphosphorylated tau, increased cell apoptosis
	Lin et al., 2018 [109]	cerebral organoids	CRISPR/Cas9-generated isogenic iPSC lines homozygous for APOE4 alleles	Increased A*β* accumulation and tau phosphorylation

Parkinson's disease (PD)	Kim et al., 2019a [34]	Midbrain organoids	CRISPR/Cas9-generated isogenic iPSC lines harboring LRRK2 G2019S mutation	Shortened neurite length and decreased marker expression of mDAN; increased aggregation and abnormal clearance of *α*-synuclein; inhibition of upregulated TXNIP ameliorated mutant phenotypes
	Wulansari et al., 2021 [114]	Midbrain organoids	CRISPR/Cas9-mediated homozygous knockout of DNAJC6 in hESCs	mDAN degeneration, *α*-synuclein aggregation, increased neuronal firing frequencies, mitochondrial and lysosomal defects

Huntington's disease (HD)	Conforti et al., 2018 [133]	Cerebral organoids	Patient iPSC-derived	Defective progenitor identity acquisition, abnormal neuronal specification, and disrupted cellular organization
	Zhang et al., 2019 [134]	Cerebral organoids	Patient iPSC-derived and isogenic HD hESC-derived	Impaired cell cycle, disrupted neuroepithelial structures, and premature neurogenesis

Glioblastoma	Linkous et al., 2019 [139]	Cerebral organoid glioma (GLICO)	Patient-derived glioma stem cells cocultured with hESC-derived cerebral organoids	Rapid and deep invasion of glioblastoma cells into cerebral organoids; invasive tumor phenotypes in hybrid organoids
	Jacob et al., 2020b [143]	Glioblastoma organoids	Patient-derived	Recapitulated histological, cellular, and transcriptomic features of glioblastoma; aggressive infiltration after transplantation

hPSC: human pluripotent stem cell, including hiPSC and hESC; hiPSC: human-induced pluripotent stem cell; hESC: human embryonic stem cell; VZ: ventricular zone; oSVZ: outer subventricular zone; NPC: neural progenitor cells; ChP: choroid plexus; DS: Down syndrome; mDAN: midbrain dopaminergic neuron.
